# Specific IgE and IgG4 Profiles of House Dust Mite Components in Allergen-Specific Immunotherapy

**DOI:** 10.3389/fimmu.2021.786738

**Published:** 2022-02-07

**Authors:** Lin Yang, Yaqi Yang, Qingxiu Xu, Wei Zhang, Qing Jiang, Wenjing Li, Yin Wang, Dongxia Ma, Xiaomin Lin, Baoqing Sun, Rongfei Zhu

**Affiliations:** ^1^ Department of Allergy, Tongji Hospital, Tongji Medical College of Huazhong University of Science and Technology, Wuhan, China; ^2^ National Respiratory Medical Center, The First Affiliated Hospital, Guangzhou Medical University, Guangzhou, China

**Keywords:** allergen specific immunotherapy, house dust mite, allergen component-specific IgE, allergen component-specific IgG4, allergen immunotherapy (AIT)

## Abstract

**Background:**

Allergen immunotherapy (AIT) can induce immune tolerance to allergens by activating multiple mechanisms, including promoting IgG4 synthesis and blunting IgE production. However, the longitudinal data of sIgE and sIgG4 to allergen components during AIT are limited.

**Objective:**

We sought to investigate the persistence and evolution of sIgE and sIgG4 against house dust mite (HDM) components during AIT and explore their correlation with clinical responses.

**Methods:**

Sixty allergic rhinitis (AR) with/without asthma patients receiving AIT for HDM were enrolled in AIT group. Thirty AR patients without receiving AIT served as control group. Blood samples were collected for sIgE, sIgG4 to HDM components (*Derp 1, Derf 1, Derp 2, Derf 2, Derp 7, Derp 10, Derp 21* and *Derp 23*) assay at baseline, Month 6 and Month 18 of AIT. Combined symptom and medication scores (CSMS) were obtained accordingly.

**Results:**

In the AIT group, sIgG4 to the HDM components of *Derp 1, Derf 1, Derp 2* and *Derf 2, Derp 21* significantly increased at Month 18 compared to the baseline (36.2 U_A_/mL *vs* 158.8 U_A_/mL, 46.4 U_A_/mL *vs* 94.6 U_A_/mL, 80.5 U_A_/mL *vs* 152.3 U_A_/mL, 78.3 U_A_/mL *vs* 205.1 U_A_/mL, 42.3 U_A_/mL *vs* 59.3 U_A_/mL, all *p*<0.05), sIgE to HDM components didn’t see differences at baseline and at Month 18 (all *p*>0.05).The numbers of positive HDM component sIgE and sIgG4 increased from 4.5 to 5 and 0 to 1.5 respectively (both *p*<0.05). However, the changes of sIgE, sIgG4, sIgE/sIgG4 ratio and the numbers of positive HDM components had no correlations with the improvement of CSMS after AIT (all ρ<0.3). For the control group, the sIgE and sIgG4 did not change significantly during the observational period (all *p*>0.05).

**Conclusion:**

AIT can induce the production of sIgG4 to HDM components. However, the increased sIgG4 levels of HDM component do not correlate with the corresponding sIgE levels at baseline or with AIT response. sIgG4 to HDM components do not qualify as a biomarker to evaluate the efficacy of AIT.

## Introduction

For respiratory allergies, allergen immunotherapy (AIT) is the only etiological treatment option which can provide long-term relief of symptoms even after treatment cessation (1). The production of allergen-specific IgG4 response is regarded as a ‘protective’ factor of AIT (2, 3). During AIT, B-cells show a shift from producing the allergenic IgE to IgG4. In terms of allergen binding to effector cells (including mast cells and basophils), IgG4 competes with IgE to prevent these cells from activation and subsequent degranulation (4). Despite the potentially protective effect of IgG4, many studies failed to demonstrate the correlation of IgG4 and clinical response to AIT (5, 6).

Component-resolved diagnosis (CRD)is a method recently developed to detect IgE antibodies with the allergen components. CRD makes it possible to obtain the individual sensitization profile to a certain allergen (7, 8). It also enables us to observe the longitudinal evolution of complex sIgE and sIgG4 components’ profiles. A growing spectrum of molecules, representing single allergens of clinical relevance, have been identified and produced for commercial *in vitro* assays (9). Take house dust mite (HDM) a perennial allergen for example, it is known as a major risk factor for allergic diseases, including asthma, allergic rhinitis (AR), and atopic dermatitis (10). So far, 82 HDM allergen components have been identified (11). The sensitization profile of HDM components may correlate with disease severity and outcome (10). For example, in our previous study, we found that sensitization to both *Derp1* and *Derp2* may be a risk factor for developing asthma in HDM-AR patients (12).

The allergen sensitization profile may also have an impact on AIT. The quality of HDM allergen extracts used for AIT is usually standardized by the major components such as *Der p 1* and *Der p 2* but not all components (13). It is possible that the allergen components in HDM extracts are not well-matched with the HDM sensitization profile of an individual, which may lead to attenuated response to AIT. It has been estimated that non-responder may comprise up to 30% of patients treated with AIT (14). We hypothesize the HDM component sIgE and sIgG4 profile plays an important role in AIT. However, the data of longitudinal evolution of sIgE and sIgG4 against HDM components during AIT is limited. Some studies provided cross-sectional IgG4 data rather than longitudinal profiles. Therefore, the aim of this study was to investigate the profiles of sIgEs and sIgG4s generated against HDM components over 18 months of AIT and to assess if they could be potential biomarkers to evaluate the AIT responses.

## Methods

### Patients

The study investigated the AR patients receiving HDM subcutaneous immunotherapy from January 2017 to January 2019 in Tongji Hospital. The AR patients without receiving AIT at the same period were enrolled as control. This study was approved by the Independent Ethical Committee of Tongji Hospital, and each participant or his or her statutory guardian signed the written informed consent before receiving AIT.

Patients enrolled in our study were those who (1) were diagnosed with HDM-induced AR with/without asthma according to Allergic Rhinitis and its Impact on Asthma (ARIA) guidelines and Global Initiative for Asthma (GINA) guidelines (13, 15); (2) had positive skin prick test (SPT) to HDM and positive serum HDM specific IgE (sIgE≥0.35kU/L). The patients with upper respiratory infection or chronic rhinosinusitis were excluded. The SCIT for the children was decided and delivered by their referring doctors after a thorough communication with their parents.

### Symptom and Medication Use Assessments

Four AR symptoms (sneezing, rhinorrhea, itching, and nasal obstruction) were measured on a scale from 0 to 3 (0 = no symptom, 1 = mild, 2 = moderate, 3 = severe symptom). The average value of the four symptom scores (ranging from 0 to 3) was the final symptom score. All subjects received medications according to the ARIA and/or GINA guidelines, including nasal corticosteroids and antihistamines. The medication score was defined as 0 = “no rescue medication taken”; 1 = “use of antihistamines (oral, intranasal, ophthalmic, or both)”; 2 = “use of nasal corticosteroids”; and 3 = “use of oral corticosteroids”. The highest score was recorded for the medication score if more than 1 class of rescue medication was used on a particular day. The medication score ranged from 0 to 3. The combined symptom and medication score (CSMS) was the sum of symptom scores and medication scores (ranging from 0 to 6). CSMS was obtained at baseline, Month 6 and Month 18 of AIT (12).

### AIT

All these patients receiving subcutaneous immunotherapy followed a conventional schedule with a commercial product of ALK (AlutardSQ; ALK, Hørsholm, Denmark) or NHD [NovoHelisen Depot (NHD); Allergopharma, Reinbek, Germany] according to the manufacturer’s instructions.

### Allergen sIgE and Components sIgE and sIgG4 Test

Blood samples were collected for sIgE, sIgG4 to HDM components (*Derp 1, Derf 1, Derp 2*, *Derf 2, Derp 7, Derp 10, Derp 21* and *Derp 23)* assay at three time points (baseline, Month 6 and Month 18) for the AIT group, two time points (baseline and Month 18) for the control group. The sIgE against *Dermatophagoides pteronyssinus (Der p)* and *Dermatophagoides farinae (Der f)* were measured by ImmunoCAP-250 system (Thermo Fisher Scientific), sIgE level over 0.35 kUA/L was defined as positive. sIgE and sIgG4 of HDM components (*Derp 1, Derf 1, Derp 2* and *Derf 2, Derp 7, Derp 10, Derp 21* and *Derp 23)* were measured by HDM components sIgE and sIgG4 test kit (Hangzhou ZhedaDixun Biological Gene Engineering Co, LTD, Hangzhou, China). The recombinant HDM components (*Derp 1, Derf 1, Derp 2*, *Derf 2, Derp 7, Derp 10, Derp 21* and *Derp 23)* were precoated on the chip. After serum was incubated on the chip for 1-hour, anti-human IgE/IgG4 antibodies (conjunct by biotin) and alkaline phosphatase-streptavidin were added consecutively. The concentrations of IgE/IgG4 were calculated by a series of IgE/IgG4 protein standardizations. The positive *cut-off* value of sIgG4 was set by 95% percentile of upper limit of 210 healthy non allergic donors, sIgG4 to HDM components above 156 U_A_/mL was defined as positive. SIgE to HDM components level above 0.35 IU/mL was defined as positive.

### Competitive Inhibition Test Between sIgE and sIgG4 Against HDM Components

High-binding microtiter plates were coated overnight at 4°C with 20µg/mL antihuman IgG4 antibody solution (Southern Biotech, Bermingham, USA) and then blocked with bovine serum albumin at 1% in phosphate-buffered saline solution plus 0.05% Tween-20. Then, 100µlof diluted serum was incubated in the plates 3 times (1:1) for 3 hours at 37.0°C. The sIgE and sIgG4 of HDM components of the supernatant and the diluted serum (1:1) were measured by ZhedaDixun test kit as previously described.

### Cell Staining and Flow Cytometric Analysis

PBMCs of patients at baseline and after 18-month AIT were isolated and stored in liquid nitrogen. Thawed PBMCs were incubated with 40µg/mL HDM allergen for 5 days, then the harvested cells were prepared at a concentration of 1×10^6^ in 100µl FACS staining buffer and were stained with anti-human CD3, CD4, CD279 (PD-1), CD183 (CXCR3), CD185 (CXCR5), CD294 (CRTH2), CD25 and Foxp3 antibodies (Biolegend, USA). Stained cells were acquired with Cytek Aurora flow cytometer (Cytek Biosciences, Fremont, CA, USA) and the data were analyzed with Flowjo software (Treestar, Ashland, OR).

### Statistical Analysis

Descriptive parameters, such as means and Standard Error of Mean (Mean ± SEM) for normally distributed continuous data, frequencies, and percentages for categorical data, were calculated. The abnormal distribution data were expressed as median and 25% to 75% interquartile ranges. T-test and non-parametric Wilcoxon paired test were used for comparisons of data in normal and non-normal distributions, respectively. The Mann–Whitney U test was used to compare numerical data between groups, and the Spearman rank test was used to assess correlations. *p*<0.05 was considered statistically significant. All statistical analyses were performed with GraphPad Prism 9.0 (GraphPad Software, San Diego, California USA).

## Results

### The Characteristics of the Patients

**Figure 1:**
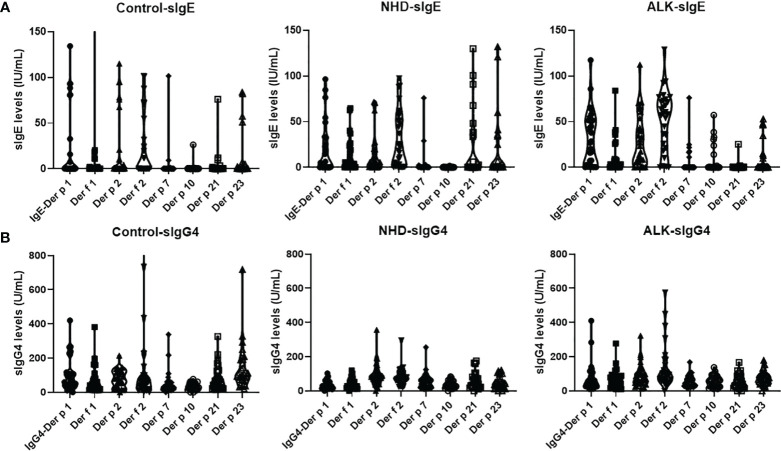
Levels of serum sIgE and sIgG4 to *Der* p 1, *Der f* 1, *Der p* 2, *Der f* 2, *Der p* 7, *Der p* 10, *Der p* 21 and *Der p* 23 allergens at baseline; **(A)**. sIgE levels of HDM components at baseline; **(B)**. sIgG4 levels of HDM components at baseline. *Der p*, *Dermatophagoidespteronyssinus*; *Der f*, *Dermatophagoidesfarinae*.

A total of 90 AR patients (30 AR patients without AIT as control, 30 AR patients treated with NHD, and 30 AR patients treated with ALK) were included. The mean age has no statistical difference within the three groups (18.5 ± 3.1 years old for the AR control group; 17.0 ± 2.6 for the NHD group; 17.5 ± 2.5 for the ALK group). Twenty-one patients had history of asthma, only two were in the control group (*p*<0.05). The mean CSMS score was 2.9 ± 0.3, 3.2 ± 0.2 and 4.1 ± 0.5 respectively at baseline. There were no significant differences in demographic characteristics in the control, NHD and ALK groups (**Table 1**). Compared to the AIT groups, the *Der p* and *Der f* tested had statistical difference in the control group (*p*<0.001). The sIgE and sIgG4 levels of HDM components at baseline were also showed in **Figure 1**. In the study, 71.1%, 60%, 77.8%, 71.1%, 23.3%, 13.3%, 32.2% and 38.9% of the 90 AR patients have positive sIgE with *Derp 1, Derf 1, Derp 2* and *Derf 2, Derp 7, Derp 10, Derp 21* and *Derp 23* respectively. Only 36.7% of patients had positive IgG4 (>156 U_A_/mL) to HDM components at baseline.

**Table 1:** Demographic data and clinical characteristics.

	Control	AIT-NHD	AIT-ALK
Age, years	18.5 ± 3.1	17.0 ± 2.6	17.5 ± 2.5
Male/Female	19/11	20/10	17/13
Atopic family history (Y/N)	9/21	12/18	10/20
Smoking history (Y/N)	5/25	6/24	7/23
AR/ARAS	28/2*	20/10	21/9
Year	6.1 ± 1.6	7.0 ± 1.3	6.9 ± 1.4
CSMS Scores	2.9 ± 0.3	3.2 ± 0.2	4.1 ± 0.5
*Der p*IgE, kU/l	1.3 (0.4-9.7)***	12.6 (3.8-92.7)	59.5 (14.0-88.2)
*Der f*IgE, kU/l	1.4 (0.5-17.4)***	19.8 (6.6-84.0)	46.0 (15.0-87.6)

Data are presented as numbers or Mean ± SEM; AR, Allergic Rhinitis; ARAS, Allergic Rhinitis with Asthma; Y/N, Yes/No. The sIgE is presented as median and 25% to 75% interquartile range (IQR). (*p < 0.05; ***p < 0.001).

### sIgE and sIgG4 of HDM Components in the Control and AIT Groups

**Figure 2:**
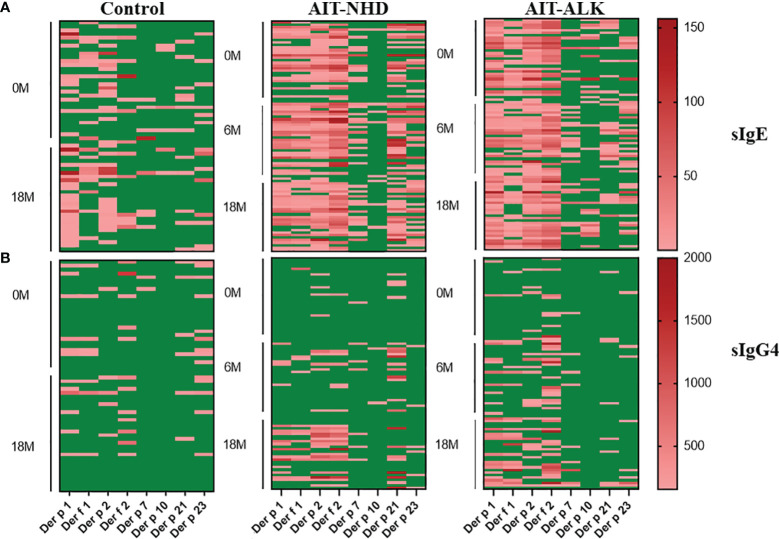
Heatmap visualization of sIgE and sIgG4 to HDM components of the 30 control patients and 60 AIT patients. Each row represents 1 patient, with green color for the concentrations below 0.35IUm/L for sIgE and 156U_A_m/L for sIgG4; red color for sIgE above 0.35IUm/L and sIgG4 above 156U_A_m/L from light red to dark red as the concentration increases. **(A)** sIgE levels at 0M and 18M in the control group; sIgE levels at 0M, 6M and 18M in the AIT groups; **(B)**. sIgG4 levels at 0M and 18M in the control group; sIgG4 levels at 0M, 6M and 18M in the AIT groups.

The longitudinal evolution of sIgE and sIgG4 against HDM components was evaluated. In the control group, both sIgE and sIgG4 against HDM components did not change at Month 18 compared to baseline (*p*>0.05). In the AIT group, the pattern of sIgE against HDM components didn’t show obvious changes during AIT; however, the sIgG4 against *Der p* 1, *Der f* 1, *Der p* 2, *Der f* 2, *Der p* 7 and *Der p* 21 significantly increased in a time-dependent manner during AIT (*p*<0.05) (**Figure 2**).

### Longitudinal Changes in sIgE, sIgG4 to HDM Components

**Figure 3:**
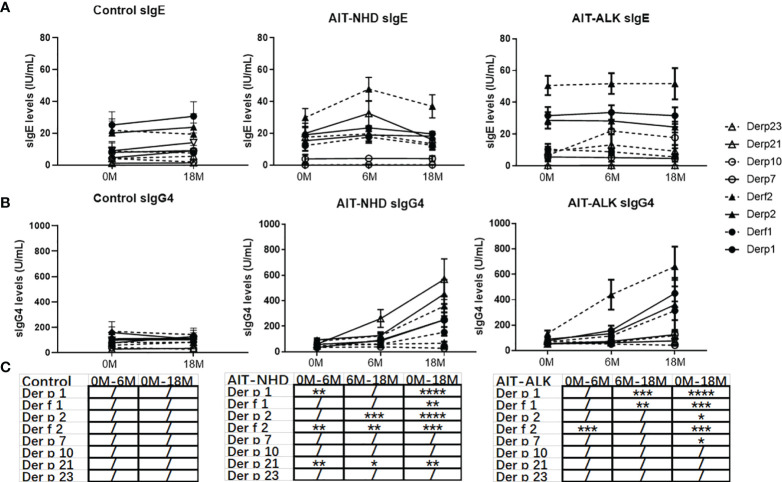
The longitudinal changes in sIgE, sIgG4 to HDM components during 18 months of AIT. **(A)** sIgE of HDM components of the control group, NHD group and ALK group; **(B)** sIgG4 of HDM components of the control group, NHD group and ALK group; **(C)** The *p* value of sIgG4 of the control group, NHD group and ALK group between 0M to 6M, 6M to 18M and 0M to 18M. (/>0.05; *p < 0.05; **p < 0.01; ***p < 0.001; ****p < 0.0001).

The trend lines of the allergenic HDM components leading to production of sIgE and sIgG4 were shown in **Figure 3**. During the 18 months, sIgE and sIgG4 of all the HDM components in the control group had no statistical difference. Compared to baseline, the levels of sIgE had no statistical changes at Month 6 and Month 18 with all the HDM components in the ALK group (*p*>0.05). However, during the course of AIT, the sIgEs against *Der f* 2 and *Derp*21 were statistically elevated during the up-dosing period (0M-6M) and decreased from 6M to 18M in the ALK group. The sIgG4 against *Der p* 1, *Der f* 1, *Der p* 2, *Der f* 2 and *Der p* 21 showed a significant increase after 18 months of AIT in the NHD group and *Der p* 1, *Der f* 1, *Der p* 2, *Der f* 2 and *Der p* 7 in the ALK group. The sIgG4 against *Der p* 1, *Der f* 2, *Der p* 21 in the NHD group and *Der f* 2 in the ALK group already increased only after 6 months of AIT.

### HDM Components Antibodies Positive/Negative Evolution During AIT

**Figure 4:**
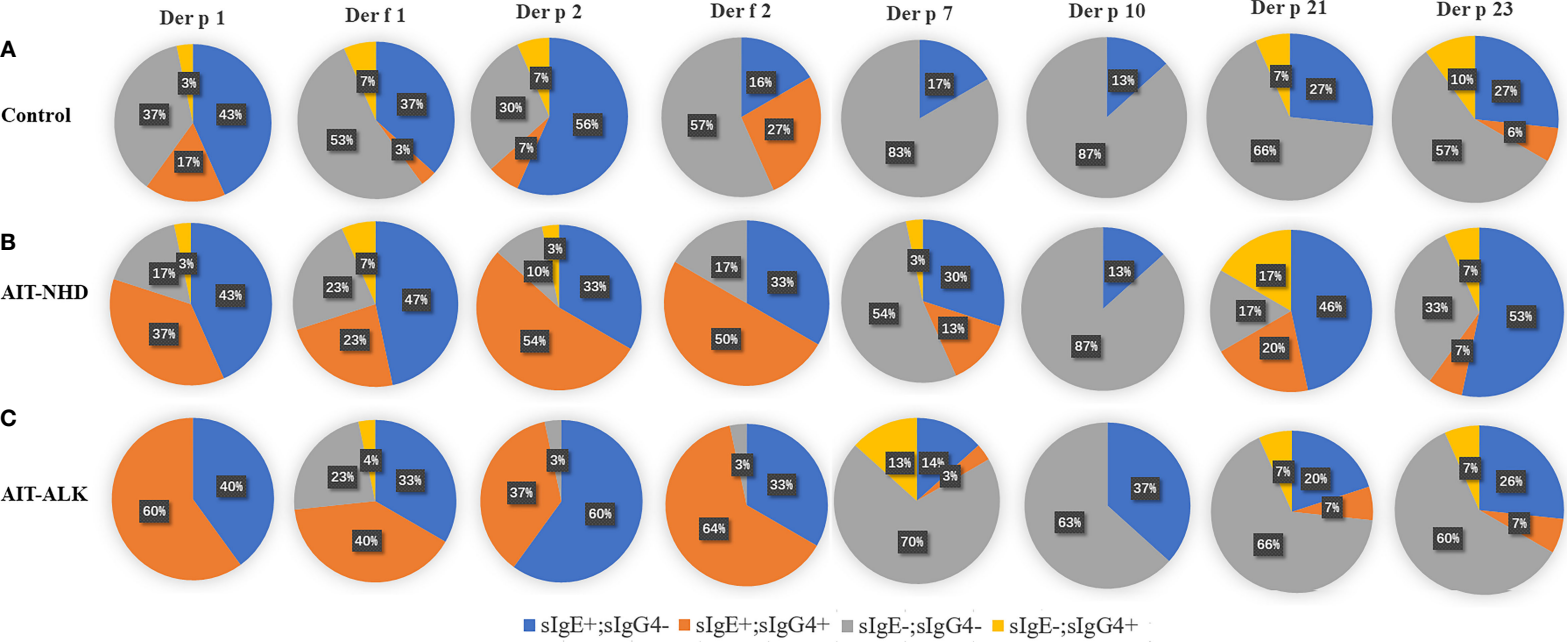
sIgE and sIgG4 sensitization patterns of HDM components. All the patients were divided into four patterns, according to the negative or positive level of sIgE at 0 month and sIgG4 at 18-month. The pie chart shows the proportion of the four patterns of the eight HDM components. **(A)** sIgE and sIgG4 pattern of HDM components in control group; **(B)** sIgE and sIgG4 pattern of HDM components in the NHD group; **(C)** sIgE and sIgG4 pattern of HDM components in the ALK group.

The positive or negative level of sIgG4 and sIgE during AIT was also assessed by qualitative analysis. There was no statistical difference of positive sIgE and sIgG4 HDM components after 18 months in the control group. However, the number of positive sIgG4 against HDM components increased statistically at Month 18 compared with baseline in the NHD group and the ALK group (both *p*<0.001). Meanwhile, the number of positive sIgE against HDM components also increased after AIT in the two groups, which showed a similar pattern as sIgG4 (both *p*<0.05) (**Table 2**). Eight HDM components sensitizations were divided into four repertoires by sIgE at baseline and corresponding sIgG4 (against the same HDM component) at Month 18 (sIgE+/sIgG4-; sIgE+/sIgG4+; sIgE-/sIgG4-; sIgE-/sIgG4+). As is shown in **Figure 4**, up to 56% of patients failed to induce sIgG4 in the control group after Month 18, which was much higher than those patients with sIgG4 production (0% to 27%). In the AIT groups, 23% to 64% patients with positive sIgE at baseline induced sIgG4 after 18 months of AIT with *Der p* 1, *Der f* 1, *Der p 2* and *Der f* 2. In contrast, more patients with positive sIgE at baseline failed to induce sIgG4 after AIT with *Der p* 7, *Der p* 10, *Der p 21* and *Der p 23*.

**Table 2:** The number of positive sIgE and sIgG4 against HDM components.

		0M	6M	18M	*p* (0-6M)	*p* (6-18M)	*p* (0-18M)
	Control	3 (1-4)	/	3 (2-4)	/	/	0.90
sIgE	NHD	4.5 (2.8-6)	6 (3-7)	6 (3.8-7)	0.042*	0.49	0.011*
	ALK	4.5 (3-5)	5 (4-5.3)	5 (4-6)	0.08	0.35	0.007**
	Control	0 (0-3)	/	0 (0-1.25)	/	/	0.58
sIgG4	NHD	0 (0-0)	0 (0-1)	1.5 (0-4)	0.054	0.0013**	<0.0001**
	ALK	0 (0-0)	1 (0-1)	1.5 (0-3)	0.0037**	0.019*	0.0005**

The number of positive sIgE or sIgG4 against HDM components were presented as median and 25% to 75% interquartile range (IQR). (*p < 0.05; **p < 0.01).

### Correlations of sIgE Levels at Baseline and sIgG4 After 18 Months of AIT

**Figure 5:**
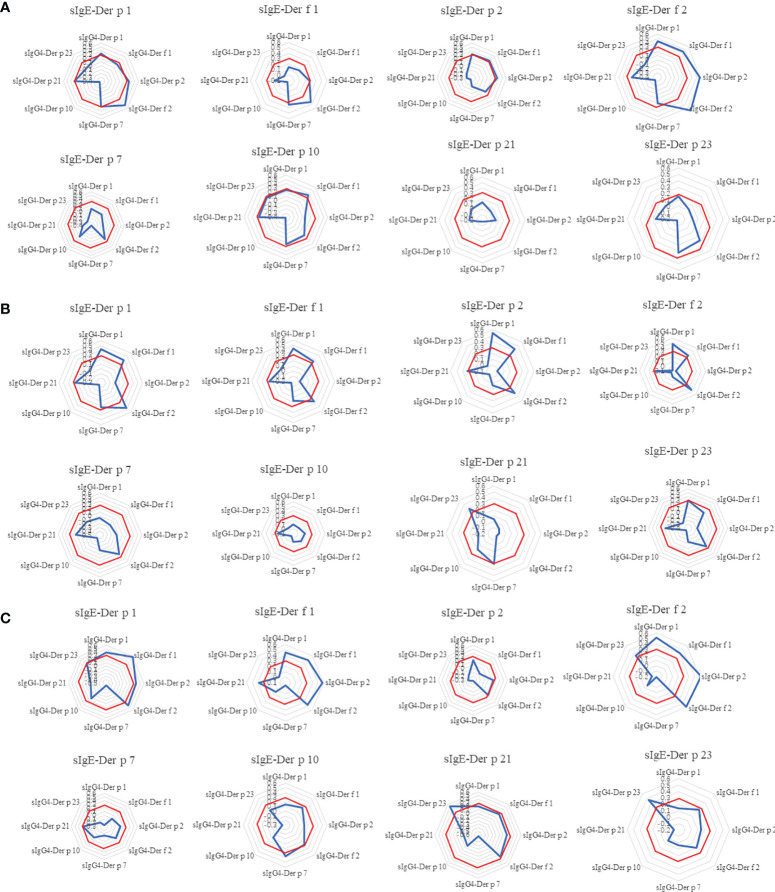
The Spearman correlation analysis between sIgE of HDM components at 0M and sIgG4 of HDM components at 18M. The radar image showed the correlation between each HDM components of sIgE at baseline and the eight components of sIgG4 at 18-month. **(A)** control group; **(B)** AIT-NHD group; **(C)** AIT-ALK group. To better illustrate the results of correlation, ρ = 0.3were labeled as red octagonal box. The ρ values in the red box indicates that there is no correlation.

IgG4 can competitively block the binding of IgE to antigen. Our study tried to look into the correlation of sIgE level at the baseline and sIgG4 levels at Month 18 of control patients and AIT patients. What interested us was the HDM components sIgG4 after Month 18 showed mild or no correlations to their corresponding component of sIgE at baseline in all the three groups (**Figure 5**). In the control group, the sIgE of *Der p1* and *Der f 1* showed mild correlation with sIgG4 of *Der f 2*. sIgE of *Der f 2* correlated with *sIgG4 of* major HDM components (*Der p 1, Der f 1, Der p 2* and *Der f 2*) (ρ>0.3, *p*<0.05). In the NHD group and ALK group, the sIgE of major HDM components at baseline had weak correlations with sIgG4 levels of major HDM component and no correlation with sIgG4 levels of *Der p* 7, *Der p* 10, *Der p 21* and *Der f* 23. Meanwhile, sIgE at baseline of *Der p* 7, *Der p* 10, *Der p 21* and *Der f* 23 had no correlation with all the sIgG4 of HDM components after AIT in most cases.

### The Capacity of IgG4 to Block Binding of IgE to HDM Allergens

**Figure 6:**
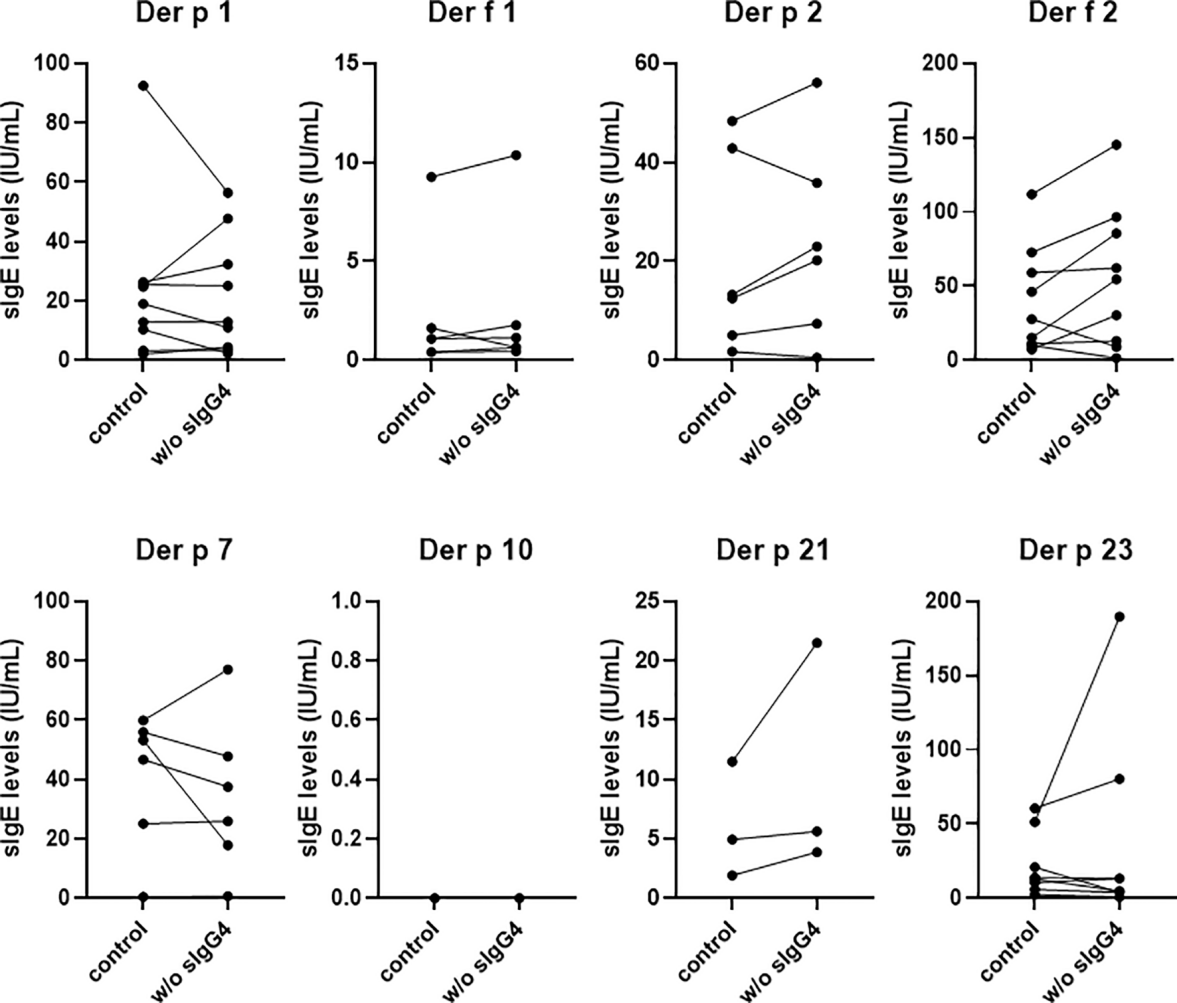
The capacity of IgG4 to block binding of IgE to HDM allergens. sIgE levels against HDM components of 9 AIT patients after 18 months. Only the positive sIgE of the eight HDM components were shown and compared here. w/o sIgG4 presents the sIgE of serum deletion of sIgG4.

For 9 randomly selected AIT patients, the IgG4 antibody was removed from the serum by anti-IgG4 antibody through ELISA. The positive sIgE levels of the eight HDM components before and after sIgG4 removal were shown in **Figure 6**. Our analysis showed that the sIgE levels of most HDM components increased after sIgG4 removal; however, there was still some HDM components whose sIgE levels decreased. Unfortunately, there was no positive *Der p* 10 sIgE in our study with the 9 selected patients to analyze.

### Correlation of Allergen-Stimulated CD4+ T Cell Subsets and HDM Components Antibodies

**Figure 7:**
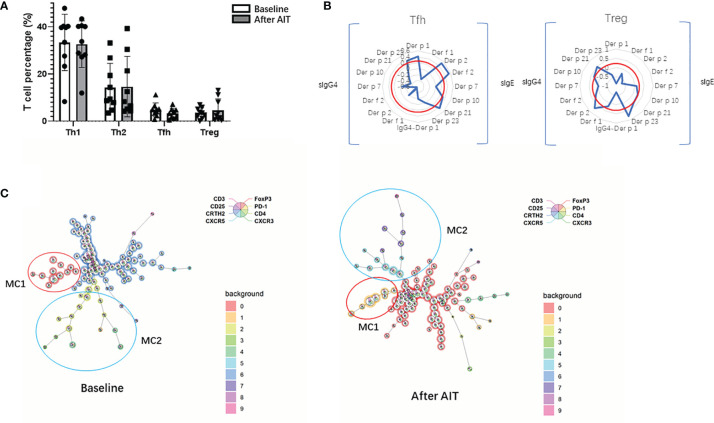
CD4+ T cell subsets and correlation with HDM components antibodies. **(A)**. Levels of Th1, Th2, Tfh and Treg at baseline and after AIT; **(B)** The Spearman correlation analysis between Tfh, Treg and HDM components antibodies. The left half part of the radar map exhibited the sIgG4 of HDM components with cells and the right part were sIgE of HDM components. ρ = 0.3were labeled as red octagonal box; **(C)** Spanning tree visualization of a self-organizing map using compensated flow cytometric data. Data were taken from the lineage (CD3^+^, CD4^+^) gate. FlowSOM nodes represent clusters of cells. Metaclusters of the nodes, determined by the map, are represented by the background color of the nodes.

The PBMCs from 9 patients were collected and incubated with 40µg/ml HDM extract for 5 days. The percentages of allergen-stimulated Th1(CD4^+^CXCR3^+^), allergen-stimulated Th2(CD4^+^CRTH2^+^), allergen-stimulated Tfh (CD4^+^CXCR5^+^PD-1^+^) and allergen-stimulated Treg (CD4^+^CD25^+^Foxp3^+^) had no significant changes after AIT. However, the changes of Tfh(ratio=baseline/after AIT) were correlated with the change of sIgE HDM components (*Der p*1, *Der p* 2, *Der f* 2, *Der p 10*and *Der f* 21) and sIgG4 *Der f* 23. Meanwhile, the change of Treg correlated with the change of sIgE HDM components (*Der p 2*1, *Der f* 23) and sIgG4 (*Der p* 2, *Der p 10* and *Der f* 21) (**Figure 7**). The FlowSOM algorithm was further used to cluster, visualize and compare the differences of CD4^+^T cells between baseline and after AIT. The FlowSOM analysis identified the metacluster1(CD3^+^CD4^+^CXCR5^+^) decreased while metacluster 2(CD3^+^ CD4^+^ CD25^+^ PD-1^+^) increased after AIT (**Figure 7**).

### Correlation Between AIT Efficacy and sIgG4, sIgE/sIgG4

**Figure 8:**
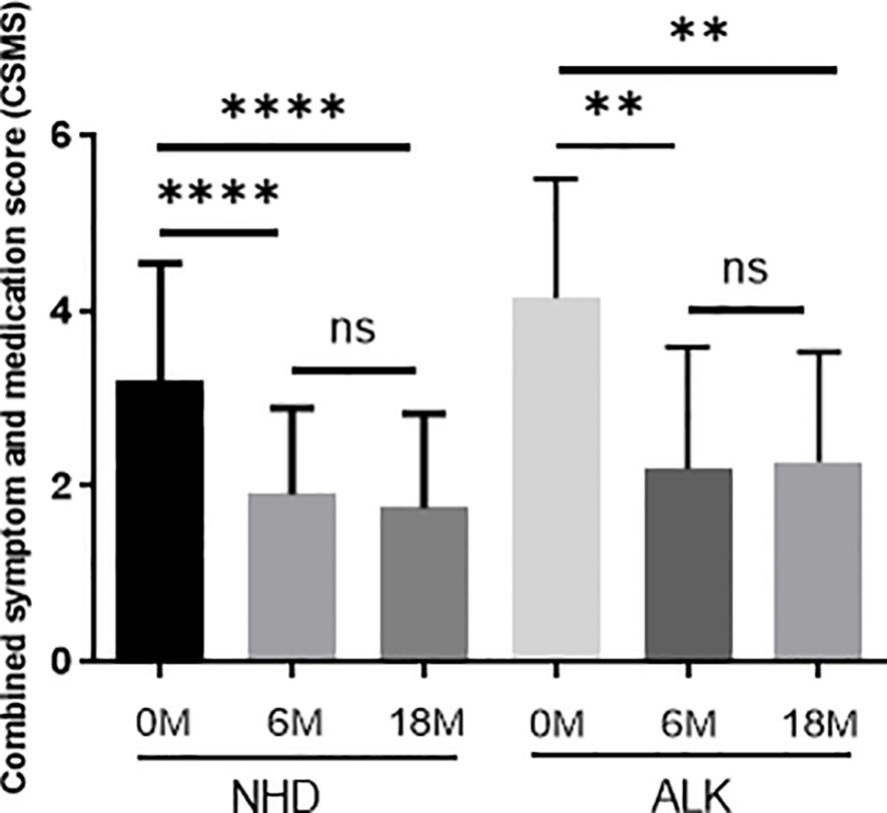
The symptom and medication scores during 18 months of AIT. The data were presented by the change from baseline (after treatment minus before treatment); the horizontal bars show the mean with 95% confidence limits. 0M=0 month; 6M = 6 months of AIT; 18M = 18 months of AIT.(ns > 0.05; **p < 0.01; ****p < 0.0001).

Both the NHD group and the ALK group showed a significant reduction in symptom and medication score (CSMS) at Month 6 and Month 18 of AIT compared to baseline (all *p*<0.05) (**Figure 8**). The △CSMS (CSMS at 18-month subtracts CSMS at baseline) exhibited no correlations with △IgG4 against HDM component (IgG4 at 18-month subtracts IgG4 at baseline) and sIgE/sIgG4 ratio at 18-month (all *p*>0.05) (**Table 3**). There were no differences of △IgG4 against HDM component and sIgE/sIgG4 ratio among the AIT responders(defined as CSMS decreased >30% compared with baseline) and non-responders as well (*p*>0.05) (data not shown).

**Table 3:** CSMS correlations with levels of sIgE and sIgG4 over time during AIT.

	ρ	P value		ρ	P value
△CSMS *vs*△IgG4 Derp1	0.13	0.45	△CSMS *vs* IgE/IgG4 Derp1	-0.16	0.35
△CSMS *vs*△IgG4 Derf1	0.01	0.96	△CSMS *vs* IgE/IgG4 Derf1	-0.09	0.61
△CSMS *vs*△IgG4 Derp2	-0.02	0.91	△CSMS *vs* IgE/IgG4 Derp2	0.01	0.96
△CSMS *vs*△IgG4 Derf2	0.03	0.85	△CSMS *vs* IgE/IgG4 Derf2	-0.05	0.76
△CSMS *vs*△IgG4 Derp7	-0.14	0.39	△CSMS *vs* IgE/IgG4 Derp7	0.15	0.37
△CSMS *vs*△IgG4 Derp10	-0.07	0.67	△CSMS *vs* IgE/IgG4 Derp10	-0.16	0.34
△CSMS *vs*△IgG4 Derp21	0.13	0.45	△CSMS *vs* IgE/IgG4 Derp21	-0.06	0.70
△CSMS *vs*△IgG4 Derp23	-0.16	0.32	△CSMS *vs* IgE/IgG4 Derp23	0.12	0.49

AIT, Allergen immunotherapy; CSMS, CSMS at 0 months; **△**IgE, sIgE at 18M-sIgE at 0M; △CSMS, CSMS at 18M-CSMS at 0M; IgE/IgG4, sIgE/sIgG4 at 18M; △IgG4, sIgG4 at 18M-sIgG4 at 0M.

## Discussion

In this study, we investigated the profile of sIgE and sIgG4 against HDM components at different time-points of AIT under real-life conditions and their correlation with AIT responses. Here we presented the data of AIT with two commercially standardized HDM extracts. We found that the levels of sIgE saw an early increase at up-dosing period and a late decrease at maintenance period while the levels of sIgG4 showed a continuous increase during 18 months of AIT. We also found the numbers of positive sIgE and sIgG4 to HDM components increased after AIT. However, the levels of sIgE and sIgG4 against HDM component didn’t show correlations with AIT responses, which implies the IgG4 against allergen components may not qualify as a biomarker to evaluate the response of AIT.

In line with other studies, we found the majority of the HDM-induced AR patients were sensitized to the HDM major components *Der f 1, Der p 1, Der p 2* and *Der f2*. Meanwhile, less than 33.3% of these patients were sensitized to *Der p 7, Der p 10, Der p 21*, which was in accord with our expectation as these 3 components had been reported as minor HDM allergens in many studies. *Der p 23* is a recently identified HDM major allergen which was supposed to cause asthmatic disease with relatively high sensitization rates (ranging from 70% to 87%) in European countries (15). However, we found only 40% of the patients were sensitized to *Der p 23* in our study. Interestingly, the sensitization rate of *Derp23* was also relatively low in another small-size Chinese population (16).The difference of *Der p 23* sensitization profile may be attributed to the HDM species in different countries, but it also reflected the different immune responses among an array of ethnic groups.

Apart from the different immune responses that may be ethnicity-related, our study of the longitudinal profiles of IgE against HDM allergens *Der p* 1, *Der f* 1, *Der p* 2, *Der f* 2, *Der p 21, and Der p 23 also* showed a similar trend of increase in the up-dosing period and decrease in the maintenance period of AIT, although only *Der f 2* and *Der p 21* have statistical difference in the NHD group. Just as several studies have demonstrated that AIT could induce a transient rise in sIgE levels before a decline and a continuing increase of sIgG4 during treatment,^14^ our findings were in line with two AIT studies in China in which the longitudinal profile of IgE against *Der p* 1 and *Der p* 2 was observed for 1 year (16, 17). Different from sIgG4 that remained stable in the control group, the levels of IgG4 against HDM components increased continuously during the 18 months of AIT, except *Der p 7, Der p 10* and *Der p 23*. In short, the longitudinal evolution of sIgE and sIgG4 against HDM components were very similar to the *Der p* and *Der f* detected by ImmunoCAP.

To further understand the significance of this evolution, we also analyzed the pretreatment IgE sensitization pattern and the molecular profile of the IgG4 responses during AIT. It was reported that B-cells show a shift from producing the allergenic sIgE to the ‘protective’ sIgG4 during AIT (18). Proliferating B-cells rearrange the constant region genes in the immunoglobulin heavy chain locus to switch from expressing IgM to others (such as IgE, IgG et. al.). Class switch produces an antibody with different effector properties, without altering its antigen specificity. One study suggested the IgE sensitization profile at baseline determined the molecular profile of the IgG4 during timothy grass pollen AIT (19). However, we didn’t find a significant correlation between sIgE against a certain HDM component at baseline and sIgG4 against the same HDM component after AIT. Interestingly, we couldn’t establish the correlations of pre-treatment IgE profile with the subsequent IgG4 responses neither in the ALK nor in the NHD group. Our result implied that the induced sIgG4 against HDM components was not restricted to the pre-treatment HDM component sIgE after AIT. We hypothesize that the mechanisms of sIgG4 generation were different after high-dose exposure to HDM through AIT. The generation of sIgG4 against specific epitopes in AIT is not only due to the class-switching of already sensitized epitopes, but also due to the emergence of new epitopes. The new epitopes may explain why there were no significant correlation between pre-treatment sIgE and post-treatment sIgG4.

It was reported that the sIgE response might expand from anoligomolecular sensitization stage to apolymolecular sensitization stage in many allergic children. This phenomenon has been defined as ‘molecular spreading’ (20). Naturally, there is always a deep concern that AIT may induce or boost new sensitization to other components of a given allergen. In our study, we found that numbers of sensitization HDM components especially the major HDM components statistically increased after AIT, both in the ALK and the NHD group. Meanwhile, the HDM components sensitization didn’t correlate with the efficacy of AIT. Our findings implied the new sensitization to allergen components during AIT was of less clinical relevance. One explanation might be the competitive allergen-binding effects from sIgG4. When IgG4 was removed from the serum, we found that more sIgE could bind to the HDM components fixed on the chip.

The production of IgE and IgG4 were intricately regulated by CD4^+^ T cells, especially allergen specific CD4^+^T cells. In our study, PBMCs of the AIT patients were incubated with HDM allergen to stimulate the CD4^+^T cells. Although unrelated CD4^+^T cells might exist, the majority of HDM stimulated-CD4+T cells were antigen specific (21). Following *in vitro* stimulation with allergen, several surface markers are specifically expressed on activated CD4+ Th cells (22). We didn’t find statistical differences of CD4^+^ T cell subsets (Th1, Th2, Tfh and Treg) after AIT. However, the changes of Tfh were correlated with sIgE and Treg correlated with sIgG4. Our results suggested that the switch of IgE to IgG4 after AIT may be due to the conversion of the subsets of CD4^+^T cells. In the FLOWSOM analysis, the cluster cells (CD3^+^CD4^+^CXCR5^+^) decreased, which could induce B-cell activation and share functional properties with Tfh cells (23). The decline of peripheral CD4^+^CXCR5^+^ cells by AIT weakens the assistance to B cells. Our study also found a cluster cell (CD3^+^ CD4^+^CD25^+^) began to express PD-1 after AIT. The effect of this cluster cell (CD3^+^ CD4^+^ CD25^+^PD-1^+^) was not reported in AIT before. However, it was identified in tumor microenvironment as PD-L1^hi^ Tregs to predict the response to PD-1/PD-L1 blockade immunotherapy (24). The effect of PD-1^hi^ Tregs in AIT will be further studied in our following work. Our results indicated that Tfh plays an important role in sIgE production and Treg correlates with sIgG4 production.

The value of sIgE and sIgG4 antibodies’ profiles in predicting the efficacy of AIT has been debated for many years. The sIgE level, sIgG4 or IgE/IgG4 ratio as biomarker of successful AIT had been proposed in many studies but always failed to be validated in other studies (25). It is believed that stratifying HDM allergic patients according to molecular sensitization profiles and molecular monitoring of AIT-induced IgG responses may enhance success of AIT (26). AIT performed with HDM extracts inducing IgG antibodies only to *Der p 1* and *Der p 2* was reported to benefit patients sensitized exclusively to these allergens (27).In our study, the CSMS were significantly improved both in the NHD and the ALK group. However, the improvement of CSMS had no correlations with HDM component sIgE, sIgG4 and sIgE/sIgG4 ratio of the eight HDM components. Meanwhile, although the profiles of IgE and IgG4 responses to AIT had some differences, the clinical efficacy in the two groups were the same. Our findings suggested the changes in sIgE and sIgG4 HDM components levels couldn’t efficiently work as biomarkers for AIT.

There were some limitations in our study. Firstly, the study population in each group was relatively small. Secondly, the observational period was relatively short (18 months) whereas AIT was suggested to last for 3 to 5 years according to the current guidelines. In addition, our study was based on the patients who finished 18-month AIT. Some patients dropped out due to less efficacy and their data were not included in the analysis, which possible slightly biased interpretation of data. Thirdly, the pattern of IgE reactivity at baseline of the control group and the two AIT groups were different. However, it may not be really important to our study, as the role of control group was not to compare with the AIT groups directly, but to investigate the changes of IgE and IgG4 patterns over a relatively long term (18 months) of natural HDM exposure. With the control group, we could ensure the data observed in the AIT groups were attributed to the treatment but not to HDM exposure. Finally, the aim of our study was to investigate the changes of IgE and IgG4 profiles during AIT, although the direct comparison between the two AIT groups may not be really important in this study, it would be interesting to know if different allergen extracts could induce similar or different antibody responses. However, the sIgE against *Der f* 2 was lower, but sIgE against *Der p* 21 was higher at baseline in the NHD group compared to the ALK group (data not shown here). We found the post-treatment sIgE and sIgG4 patterns were slightly different in the two AIT groups, but we couldn’t figure out if it was caused by the different allergen components of the two HDM extracts or by the imbalance of IgE pattern at baseline.

In summary, our study revealed longitudinal evolution of sIgE and sIgG4 against HDM components during AIT shown in an early increase and a late decrease of IgE level and a continuous increase of sIgG4 level. However, the pretreated IgE sensitization patterns couldn’t determine the molecular profile of the IgG4 responses during AIT. The post-treated sIgE and sIgG4 profiles had no correlations with AIT efficacy. Future studies combining antibodies functional binding, allergen epitope sensitization and clinical manifestations may help to elucidate the mechanism of AIT.

## Ethics approval number

TJ-IRB20210761

## Data Availability Statement

The raw data supporting the conclusions of this article will be made available by the authors, without undue reservation.

## Ethics Statement

The studies involving human participants were reviewed and approved by Medical ethics committee of Tongji Hospital, Tongji Medical College of Huazhong University of science and technology. Written informed consent to participate in this study was provided by the participants’ legal guardian/next of kin.

## Author Contributions

RZ and BS were responsible for the work as a whole. LY analyzed the HDM components data in the study and wrote the first draft of the manuscript. YY, WL, YW, and DM were responsible for CSMS data. QX and XL were responsible for data analysis. WZ and QJ contributed to IgE test by ImmunoCAP. All co-authors have contributed to the interpretation of the data and provided advice and approval of the final version of the manuscript.

## Conflict of Interest

The authors declare that the research was conducted in the absence of any commercial or financial relationships that could be construed as a potential conflict of interest.

## Publisher’s Note

All claims expressed in this article are solely those of the authors and do not necessarily represent those of their affiliated organizations, or those of the publisher, the editors and the reviewers. Any product that may be evaluated in this article, or claim that may be made by its manufacturer, is not guaranteed or endorsed by the publisher.
